# Advances in Smart Hydrogels for Myocardial Infarction Therapy: A Scoping Review of Applications, Challenges, and Future Perspectives

**DOI:** 10.1155/ijbm/7925127

**Published:** 2026-10-18

**Authors:** Eyram Ahadzi, Otema Darfour Manu, Ruth Antwi-Baah, Henry Agbe, Prince Ebenezer Adjei

**Affiliations:** ^1^ Department of Computer Engineering College of Engineering Kwame Nkrumah University of Science and Technology Kumasi 00233 Ghana knust.edu.gh; ^2^ College of Ecology and Environment Chengdu University of Technology Chengdu 610059 China cdut.edu.cn; ^3^ Department of Materials and Metallurgical Engineering College of Engineering Kwame Nkrumah University of Science and Technology Kumasi 00233 Ghana knust.edu.gh; ^4^ Global Health and Infectious Diseases Group Kumasi Centre for Collaborative Research in Tropical Medicine Kwame Nkrumah University of Science and Technology Kumasi 00233 Ghana knust.edu.gh; ^5^ Global One Health Research Group Bernhard Nocht Institute for Tropical Medicine Hamburg 20359 Germany bni-hamburg.de

**Keywords:** cardiac tissue regeneration, cardiovascular diseases, myocardial infarction, myocardial regeneration, smart hydrogels

## Abstract

Smart hydrogels are stimuli‐responsive biomaterials that enable targeted, sustained, and controlled therapeutic delivery. Their unique ability to respond to physiological cues such as pH, temperature, reactive oxygen species (ROS), and enzymatic activity makes them highly attractive for myocardial infarction (MI) therapy. This scoping review explores advances, applications, challenges, and future perspectives related to the use of smart hydrogels in MI. An initial comprehensive search of PubMed, Scopus, and ScienceDirect yielded 1802 records, from which 32 studies published between 2011 and 2025 met the inclusion criteria under the PRISMA‐ScR framework. An updated search identified seven additional eligible studies, bringing the total to 39 included studies. These studies primarily focused on smart hydrogels for delivering bioactive agents, including growth factors, stem cells, exosomes, and nanoparticles, to enhance myocardial regeneration and repair. Evidence from preclinical models consistently demonstrated improvements in myocardial remodeling, angiogenesis, cell survival, and functional cardiac recovery. Despite these encouraging findings, several important limitations were identified. Studies relied on short evaluation periods, lacked long‐term follow‐up, and were restricted to small‐animal models, thereby limiting the robustness of translational insights. Large‐animal studies and early clinical investigations remain scarce, highlighting the gap between experimental progress and clinical application. Nonetheless, smart hydrogels stand out as a promising therapeutic platform because of their capacity for localized, adaptive, and sustained responses within the complex postinfarct microenvironment. Future work should prioritize long‐term efficacy studies, development of minimally invasive delivery methods, and the use of clinically relevant preclinical models to better facilitate translation to human MI therapy.

## 1. Introduction

Myocardial infarction (MI), commonly known as a heart attack, remains the leading cause of mortality worldwide [1–3]. It results from the obstruction of the coronary arteries, leading to a deprivation of oxygen and the irreversible death of cardiomyocytes [4, 5]. The abrupt loss of blood supply activates a cascade of inflammatory responses, fibrotic remodeling of the myocardium, and impaired cardiac function, often leading to heart failure [1, 3, 6, 7].

Current therapeutic strategies, including coronary artery bypass grafting (CABG), percutaneous coronary intervention (PCI) [3, 8], and pharmacological interventions such as anticoagulants [9], primarily aim to restore perfusion but do not facilitate myocardial regeneration [3, 10, 11]. While heart transplantation and left ventricular assist devices (LVADs) offer an alternative option for end‐stage heart failure [2, 12], their use is limited by donor organ scarcity, immunological challenges [13–15], and limited accessibility [12]. Moreover, the adult myocardium exhibits low regenerative potential due to the limited proliferation of cardiomyocytes [16, 17].

Biomaterials have emerged as promising platforms for cardiac repair [18]. Among these, hydrogels have gained considerable attention. Hydrogels are three‐dimensional networks of hydrophilic polymers that can absorb and retain large amounts of water [3, 19]. Conventional hydrogels are extensively used in biomedical applications but often suffer from inadequate mechanical strength [19–22], slow response rates, and poor elasticity [23], which limits their effectiveness in dynamic physiological environments.

To address these limitations, smart hydrogels, also known as intelligent or stimuli‐responsive hydrogels, have been developed. These materials undergo reversible chemical or physical changes in response to specific stimuli [3, 24–26], including physical (temperature, light, pressure), chemical (pH, ionic strength), or biological (antigens, enzymes, hypoxia) factors [22, 27, 28]. This stimulus responsiveness enables selective release of therapeutic agents in response to pathological cues [29], through changes in surface charge, gel volume, or bond cleavage [30], providing both fine‐tuning and spatial control of drug‐release kinetics [27, 31]. In addition, their ability to modulate physicochemical properties allows them to closely mimic the extracellular matrix (ECM) [27]. Leveraging these capabilities, smart hydrogels have gained use in tissue engineering, regenerative medicine, and targeted drug delivery [22, 32–38]. In MI therapy, they can respond to the dynamic postinfarction cues, such as oxidative stress and pH changes, enabling localized, dynamic treatment [1, 9]. By integrating structural support with controlled therapeutic delivery, smart hydrogels represent a promising approach for addressing multiple challenges associated with myocardial repair.

While several reviews have explored the role of smart hydrogels in MI therapy, they often do so within broader hydrogel systems or a general tissue engineering context [1–3, 7, 11, 22, 27, 39–42]. Some reviews have addressed cardiac tissue engineering but provide limited discussion of MI‐specific applications [32], whereas others examine smart biomaterials more broadly, with smart hydrogels representing only one component of their scope [12]. Furthermore, reviews on hydrogel‐mediated drug delivery have primarily focused on controlled release mechanisms rather than broader smart hydrogel applications and translational considerations in MI therapy [43]. Therefore, a dedicated MI‐focused synthesis integrating smart hydrogel design strategies, therapeutic applications, current challenges, and future directions remains limited.

Unlike previous reviews that have primarily examined specific aspects of hydrogel‐based cardiac repair, this scoping review maps the current landscape of smart hydrogels developed for MI therapy. Specifically, it evaluates (i) smart hydrogel platforms, including natural, synthetic, and hybrid systems, and their associated stimulus‐responsive mechanisms; (ii) cargo delivery strategies involving bioactive agents such as cells, extracellular vesicles (EVs), drugs, proteins, genetic materials, and nanoparticles; and (iii) therapeutic applications, challenges, and future directions for clinical translation. By synthesizing current evidence on smart hydrogel technologies, their therapeutic applications, and translational barriers, this review provides an MI‐focused perspective on their potential role in myocardial repair and the path toward clinical translation.

## 2. Methods

### 2.1. Review Question

Guided by the Population–Concept–Context (PCC) framework [44], this review addresses the following question: What advancements have been made in the use of smart hydrogels for MI therapy, and what are their applications, reported challenges, and future directions?

### 2.2. Search Strategy

This scoping review was conducted following the Preferred Reporting Items for Systematic Reviews and Meta‐Analyses extension for Scoping Reviews (PRISMA‐ScR) guidelines [45] (See Supporting 1). A comprehensive search was carried out across three electronic databases: PubMed, Scopus, and ScienceDirect, on February 19, 20, and 22, 2025, respectively. The search strategy employed a combination of Boolean operators (“AND” and “OR”) to link relevant keywords related to “smart hydrogels,” “myocardial infarction,” “applications,” “challenges,” and “future perspectives.” The strategy was further refined in consultation with an experienced academic librarian (SA), and the detailed database‐specific search strategies and Boolean queries are provided in Supporting 2. A final search update was conducted on 3 July 2025 to capture any newly published or previously missed studies.

### 2.3. Inclusion and Exclusion Criteria

Studies were considered eligible for inclusion if they were original research articles focused on smart hydrogels for MI therapy and published in English in peer‐reviewed journals, with no limitations on publication date. Eligible studies specifically addressed either the applications, challenges, and/or future perspectives of smart hydrogels in the context of MI therapy. Exclusion criteria comprised studies without full‐text availability, book chapters, conference abstracts, editorials, and opinion pieces. These criteria were selected to ensure the review captured high‐quality, evidence‐based literature that directly contributes to mapping the landscape of smart hydrogel research for MI therapy.

### 2.4. Study Selection

Title and abstract screening of all retrieved articles was conducted independently by two reviewers (E.A. and O.D.M.) using Rayyan (Qatar Computing Research Institute [QCRI], Qatar). Conflicts were resolved through discussion with a third reviewer (P.E.A.) before full‐text screening. Subsequently, full‐text articles meeting the inclusion criteria were assessed for eligibility and selected for data extraction by E.A. and O.D.M.

### 2.5. Data Extraction

A standardized data extraction form was developed using Microsoft Excel to chart relevant information from each included study. Extracted data comprised of the study characteristics (title, authors, year of publication, name of publication, and country), hydrogel characteristics (classification by stimuli responsiveness source and bioactive agents), study design (study model/experimental model, mode of MI induction, sex, weight, strain, number of in vivo experimental groups, assessment time points, delivery platform, and mode of delivery), applications, challenges, and future directions. The form was piloted on a subset of studies to ensure consistency and clarity. Data charting was performed in duplicate by two reviewers (E.A. and O.D.M.), with discrepancies resolved through consensus or consultation with a third reviewer (P.E.A.). Given the scoping design, no quantitative analysis was performed; instead, descriptive, narrative, and thematic synthesis were used to summarize findings, map trends, and highlight applications, challenges, and future directions in smart hydrogel therapy for MI.

#### 2.5.1. Use of Artificial Intelligence (AI) Tools

AI tools, including ChatGPT and Grok, were used solely to improve the academic language, clarity, and readability of the manuscript. All intellectual content, analyses, interpretations, and conclusions were developed by the authors.

## 3. Results

### 3.1. Study Selection and Characteristics

The PRISMA‐ScR flow diagram detailing the study selection process is presented in Figure 1.

**FIGURE 1:**
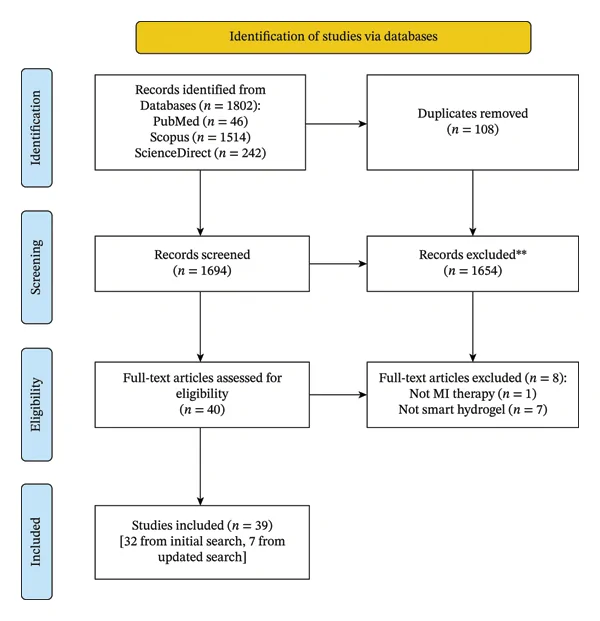
PRISMA‐ScR flow diagram of the study selection process. Created by the authors using Draw.io (diagrams.net).

The initial search yielded a total of 1802 records from PubMed (*n* = 46), Scopus (*n* = 1514), and ScienceDirect (*n* = 242). After manually removing duplicates, 1694 articles remained. Following title and abstract screening, 40 articles were selected for full‐text screening. Of these, eight articles were excluded based on the predefined eligibility criteria. As a result, 32 articles were initially included in this scoping review. The second and final search identified 2046 articles, of which seven additional studies met the inclusion criteria. In total, 39 studies were included in the scoping review. The majority of studies originated from China (*n* = 33), with additional contributions from the USA (*n* = 3) [46–48], Germany (*n* = 1) [49], and Iran (*n* = 2) [50, 51]. Most studies were published between 2020 and 2025, with only four studies published between 2011 and 2019 [23, 46, 47, 52].

### 3.2. Hydrogel Characteristics

#### 3.2.1. Classification by Stimuli

Among the 39 studies, temperature was the only reported physical stimulus in the context of MI therapy, appearing in eight studies [6, 49, 51, 53–57]. Chemically responsive hydrogels primarily targeted reactive oxygen species (ROS), as observed in 12 studies [58–69], while Cheng et al. targeted pH sensitivity [70]. Biologically, enzymatic responsiveness was explored in four studies, all targeting matrix metalloproteinases (MMPs) [46, 52, 71, 72], commonly upregulated in postinfarction tissue remodeling [73–76]. Notably, one study incorporated responsiveness to transglutaminase [77]. Several smart hydrogels demonstrated dual‐stimuli responsiveness and were reported in 13 studies (see Table 1). Six studies used hydrogels that responded to both pH and ROS [31, 78–80, 84, 85]. One each responded to temperature and ROS [5], temperature and enzyme [82], ROS and light [83], pH and temperature [47], enzyme and hypoxia [48], and temperature and electrical stimulation [50]. Wang et al. [81] and Xu et al. [65] each reported hydrogels responsive to both ROS and hypoxia. These stimulus‐responsive strategies enable hydrogels to adapt to the infarct microenvironment, as summarized in Figure 2.

**TABLE 1:** Summary of included studies: hydrogel source, stimuli responsiveness, and encapsulated agents.

Stimuli	Source	Bioactive agent	Reference
ROS	Hybrid	None	He et al. [58]
MMP	Natural	GST‐TIMP‐bFGF	Fan et al. [52]
ROS, pH	Hybrid	Curcumin, rhCol III, RepSox	Liu et al. [78]
ROS, pH	Hybrid	Curcumin, rhCol III	Hu et al. [79]
Temperature	Natural	AC16 cell‐derived EVs	Hua et al. [53]
ROS, pH	Hybrid	Dexamethasone	Wang et al. [80]
pH	Hybrid	Metformin, MSC‐exosomes	Cheng et al. [70]
ROS	Hybrid	MCT nanoparticles	Xu et al. [59]
Temperature	Hybrid	EGCG	Wang et al. [54]
Temperature	Hybrid	hiPSC‐cardiomyocytes	Roshanbinfar et al. [49]
Hypoxia, enzyme	Hybrid	Stromal cell‐derived factor‐1 alpha, oxygen‐releasing microparticles	Hassan et al. [48]
ROS, hypoxia	Hybrid	MSCs‐derived exosomes, CAT	Wang et al. [81]
ROS, temperature	Hybrid	BPNSs	Qiu et al. [5]
Temperature	Synthetic	Triptolide	Wang et al. [6]
ROS, pH	Hybrid	Rosmarinic acid	Zhang et al. [31]
MMP	Hybrid	IL4‐pDNA‐composited carbon dot (CTL4)	Chen et al. [71]
Temperature, electrical stimulation	Hybrid	Gold NPs, oxidized bacterial cellulose nanofibers	Tohidi et al. [50]
MMP	Hybrid	Deferoxamine	Wei et al. [72]
Temperature	Natural	MSCs	Peng et al. [55]
ROS	Natural	Basic fibroblast growth factor	Fu et al. [60]
ROS	Hybrid	rBMSCs, puerarin	Liu et al. [61]
ROS	Natural	Melanin NPs	Zhou et al. [62]
Temperature	Synthetic	Niosomal rosuvastatin	Malektaj et al. [51]
Transglutaminase	Hybrid	Salvianolic acid B	Chen et al. [77]
ROS	Synthetic	None	Liu et al. [63]
Temperature, enzyme	Natural	Human MSCs	Hua et al. [82]
ROS, light	Synthetic	CAT	Ding et al. [83]
Temperature	Natural	Umbilical cord MSCs	Ke et al. [56]
MMP‐2	Hybrid	siMMP2	Wang et al. [46]
pH, temperature	Synthetic	bFGF	Garbern et al. [47]
ROS, pH	Hybrid	CNP, Sema3A	Yang et al. [84]
Temperature	Natural	Ca^2+^	Li et al. [57]
ROS, pH	Hybrid	CsA	Zhang et al. [85]
ROS	Hybrid	bFGF	Zheng et al. [64]
ROS	Hybrid	AD‐MSC‐derived exosomes, CAT, AuNPs	Xu et al. [65]
ROS, hypoxia	Synthetic	Apelin‐13, Y1‐Gal	Zhen et al. [66]
ROS	Hybrid	FT011, S1QEL1.1, tannic acid	Luo et al. [67]
ROS	Hybrid	miR‐19a	Wang et al. [68]
ROS	Hybrid	NPs containing EGCG, hyperoside, L‐arginine	Wu et al. [69]

*Note:* AC16, human ventricular cardiomyocyte cell line; AuNPs, gold nanoparticles; BPNSs, black phosphorus nanosheets; CAT, catalase; CsA, cyclosporine A; CTL4, IL4‐pDNA‐composited carbon dot; EGCG, epigallocatechin gallate; FT011, fibrosis inhibitor FT011; GST‐TIMP‐bFGF, glutathione S‐transferase–tissue inhibitor of metalloproteinases–basic fibroblast growth factor fusion protein; IL4‐pDNA, interleukin‐4 plasmid DNA; MCT, tannic acid‐coated Mn‐Co_3_O_4_ nanoparticles; miR‐19a, microRNA‐19a; NPs, nanoparticles; RepSox, transforming growth factor‐β receptor I (TGF‐βRI) inhibitor; rhCol III, recombinant human collagen Type III; Sema3A, semaphorin‐3A; siMMP2, small interfering RNA targeting MMP‐2.

Abbreviations: AD‐MSCs, adipose‐derived MSCs; bFGF, basic fibroblast growth factor; CNP, C‐type natriuretic peptide; EVs, extracellular vesicles; hiPSC, human‐induced pluripotent stem cell; MSCs, mesenchymal stem cells; rBMSCs, rat bone marrow MSCs.

**FIGURE 2:**
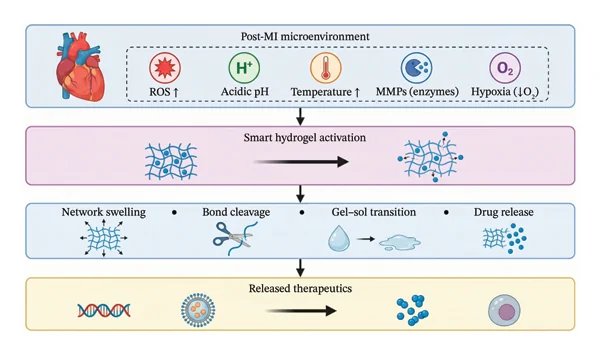
Stimulus‐responsive mechanisms of smart hydrogels in myocardial infarction therapy. Initial schematic generated using ChatGPT (OpenAI; accessed 2026), followed by AI‐assisted regeneration and refinement using BioRender. Final figure reviewed and refined by the authors for scientific accuracy. Created in BioRender. Ahadzi, E. (2026) https://BioRender.com/pppi8ui.

#### 3.2.2. Classification by Source

Hydrogels were classified into three categories based on their source: natural, synthetic, and hybrid [86–88]. Natural hydrogels were used in eight studies [52, 53, 55–57, 60, 62, 70, 82]. Synthetic hydrogels were featured in six studies [6, 47, 51, 63, 66, 83]. Additionally, hybrid hydrogels, composed of both natural and synthetic materials, were reported in 25 studies [5, 31, 46, 48–50, 54, 58, 59, 61, 64, 65, 67–72, 77–81, 84, 85].

#### 3.2.3. Bioactive Agents

Bioactive agents incorporated into stimuli‐responsive hydrogels for MI repair are categorized as cellular components, small molecules/drugs, EVs, protein‐based therapeutics, genetic materials, and nanoparticles (Figure 3). For clarity, we report the main bioactive agent in cases where multiple agents were co‐delivered. Table 1 provides a comprehensive list of all bioactive agents. Cellular components include mesenchymal stem cells (MSCs) in Peng et al. [55], Hua et al. [82], and Ke et al. [56], rat bone marrow MSCs (rBMSCs) in Liu et al. [61], and human‐induced pluripotent stem cell‐derived (hiPSC) cardiomyocytes (hiPSC‐CMs) in Roshanbinfar et al. [49]. EVs include AC16 cell‐derived EVs in Hua et al. [53], MSC‐derived exosomes in Wang et al. [54], and adipose‐derived MSC (AD‐MSC) exosomes in Xu et al. [65]. Small molecules and drugs comprise triptolide [6], rosmarinic acid [31], niosomal rosuvastatin [51], epigallocatechin gallate (EGCG) [54, 69], calcium ions(Ca^2+^)‐loaded liposomes [57], caffeic acid [58], FT011 (antifibrotic agent) [67], metformin [70], deferoxamine [72], salvianolic acid B [77], curcumin [78, 79], dexamethasone [80], and cyclosporine A (CsA) [85]. Protein‐based therapeutics comprised basic fibroblast growth factor (bFGF) in Garbern et al. [47], Fu et al. [60], and Zheng et al. [64]. Hassan et al. used stromal cell‐derived factor‐1 alpha (SDF‐1$\left.a\right)$ [48], and Ding et al. used catalase [83]. C‐type natriuretic peptide (CNP) was used by Yang et al. [84], and Apelin‐13 was used by Zhen et al. [66].

**FIGURE 3:**
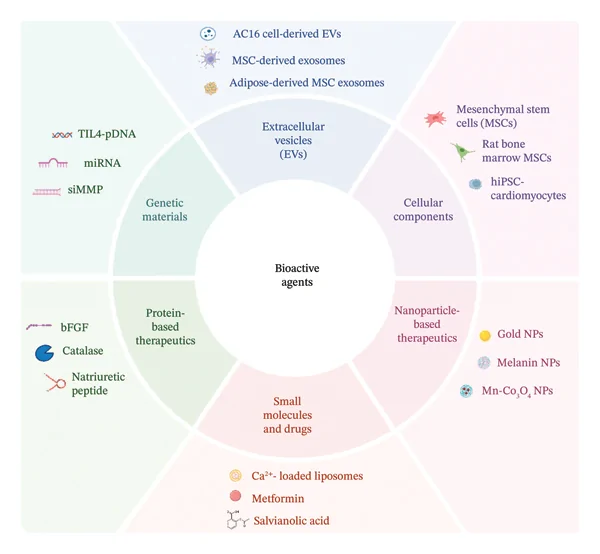
Bioactive agents delivered using smart hydrogels. Adapted from the BioRender template “Nanotherapeutics in Cancer” by Wendy Jiang, substantially modified and annotated by the authors. Created in BioRender. Ahadzi, E. (2026) https://BioRender.com/7zw7upc.

Genetic materials included small interfering MMP 2 (siMMP‐2) in Wang et al. [46], microribonucleic acid‐19a (miRNA‐19a) in Wang et al. [68], and interleukin‐4 plasmid deoxyribonucleic acid (IL4‐pDNA) composited with carbon dots in Chen et al. [71].

Nanoparticle‐based therapeutics were used in studies, including black phosphorus nanosheets (BPNSs), by Qiu et al. [5], tannic acid‐coated Mn‐Co_3_O_4_ nanoparticles by Xu et al. [59], gold nanoparticles (AuNPs) by Tohidi et al. [50], and melanin nanoparticles by Zhou et al. [62]. Interestingly, two studies attributed therapeutic effects solely to the hydrogel, without incorporating exogenous bioactive agents [58, 63].

#### 3.2.4. Hydrogel Formulation (Injectable/Scaffold‐Based)

Injectable hydrogels were the primary delivery strategy employed across the included studies. This approach facilitated the direct delivery of agents into the infarct zone, promoting localized therapeutic effects. Notably, only one study reported the use of a scaffold‐based Janus hydrogel [58].

### 3.3. Study Design

#### 3.3.1. Study Type and Experimental Models

All studies employed both in vivo and in vitro models to evaluate smart hydrogel therapies for MI, except Garbern et al. [47], which used only an in vivo model, and Tohidi et al. [50], Malektaj et al. [51], and Ke et al. [56], which focused solely on in vitro models. Among the in vitro studies, the most frequently used cell types were H9C2 (rat cardiomyoblasts) and human umbilical vein endothelial cells (HUVECs). Ralph Activated White (RAW) 264.7 macrophages were used in six studies [6, 58, 59, 69, 79], and 3T3 fibroblasts were used in five studies [46, 52, 55, 56, 79].

Fourteen studies reported the use of fibroblasts, including cardiac, NIH 3T3, and L929 fibroblasts [31, 46, 49, 52, 55, 60, 63, 65, 67, 69, 71, 72, 78, 81]. Wang et al. [80] and Yang et al. [84] used AC16 cardiomyocytes, whereas Tohidi et al. utilized human embryonic stem cell‐derived cardiomyocytes (hESC‐CMs) [50]. Roshanbinfar et al. employed hiPSC‐CMs [49]. In vivo experiments were primarily conducted using rodent models. Most studies used male Sprague‐Dawley (SD) rats weighing between 200 and 280 g [5, 53, 54, 58, 60, 61, 63–65, 67, 68, 70, 72, 80, 81, 84, 85]. Li et al. used an equal number of male and female SD rats [57]. Two studies used male Fischer rats [47, 53]. Roshanbinfar et al. used female CD1 mice [49], and another study employed C57BL/6J mice [66]. The reported ages of mice across studies ranged from 6 to 12 weeks. Most studies used animals around 6–8 weeks old [5, 58, 60, 61, 63, 65, 66, 81], one study reporting 8–10 weeks old [47], and another study using 10–12‐week‐old mice [49]. In addition to rodent models, Wang et al. used Bama minipigs weighing 30–35 kg for in vivo experimentation [68]. Animals were mostly divided into 4–6 groups, typically including sham, MI control, and hydrogel‐treated groups, and were mainly induced via left anterior descending (LAD) ligation of the coronary artery [89]. The experimental models and hydrogel delivery strategies are summarized in Table 2.

**TABLE 2:** Overview of experimental models and hydrogel delivery strategies.

Characteristic	Summary of findings
In vitro models	Commonly used models included H9C2 cardiomyoblasts, HUVECs, cardiac fibroblasts, RAW264.7 macrophages, AC16 cardiomyocytes, primary cardiomyocytes, MSCs, and hiPSC‐derived cardiomyocytes
Animal models	In vivo studies mainly used male SD rats, with occasional use of Fischer rats, CD1 mice, C57BL/6J mice, and porcine models
MI induction	LAD coronary artery ligation was the predominant induction method, with some studies using MI/R models
Delivery route	Intramyocardial injection was most common, with other approaches including direct epicardial application, intramuscular injection, and subcutaneous implantation
Animal age	Reported ages varied across models, with most studies using animals between 6 and 10 weeks old
Animal weight	Weights varied depending on the animal model, with most rat studies reporting 200–280 g

*Note:* Data synthesized from the studies included in this review [5, 6, 31, 46–72, 77–85]. AC16, human ventricular cardiomyocyte cell line; H9C2, rat cardiomyoblast cell line; RAW264.7, murine macrophage cell line; SD, Sprague–Dawley.

Abbreviations: hiPSC, human‐induced pluripotent stem cells; HUVECs, human umbilical vein endothelial cells; LAD, left anterior descending; MI, myocardial infarction; MI/R, myocardial ischemia/reperfusion; MSCs, mesenchymal stem cells.

#### 3.3.2. Mode of Hydrogel Delivery

Intramyocardial injection was the most frequently adopted approach, employed in 29 studies [5, 6, 31, 46–49, 52, 54, 55, 59–61, 64–71, 77–80, 82–85]. Hua et al. employed intramuscular injection [53], while Li et al. and Liu et al. used subcutaneous injection [57, 63]. Wei et al. combined subcutaneous and intramyocardial injections [72], while Zhou et al. used subcutaneous implantation alongside intramyocardial injection [62].

#### 3.3.3. Methods

Hydrogels were characterized for their physicochemical, mechanical, and biological properties using standard in vitro techniques. Microstructure and composition were assessed using scanning electron microscopy (SEM), transmission electron microscopy (TEM), Fourier transform infrared spectroscopy (FTIR), and proton nuclear magnetic resonance (^1H NMR). Particle size and surface characteristics were evaluated using dynamic light scattering (DLS) and zeta potential measurements, while optical properties were assessed using ultraviolet–visible spectroscopy (UV–vis).

Biological effects were assessed using a variety of in vitro assays. Cell viability and proliferation were measured using the cell counting kit‐8 (CCK‐8), 3‐(4,5‐dimethylthiazol‐2‐yl)‐2,5‐diphenyltetrazolium bromide (MTT) assay, and live/dead staining. Oxidative stress and ROS were evaluated using assays such as 2′,7′‐dichlorofluorescin diacetate (DCFH‐DA) staining, while migration and angiogenic potential were assessed using scratch assays and tube formation assays. Flow cytometry, immunofluorescence (IF), enzyme‐linked immunosorbent assay (ELISA), quantitative real‐time polymerase chain reaction (qRT‐PCR), and Western blotting (WB) were used to investigate cellular responses and molecular mechanisms.

In vivo assessments included echocardiography for cardiac function evaluation, histological staining approaches such as hematoxylin and eosin (H&E) and Masson’s Trichrome for tissue remodeling, fibrosis, apoptosis, and infarct size analysis. Some studies further evaluated functional integration through assessments of electrical conduction, hemodynamic changes, and arrhythmia susceptibility. A full list of reported evaluation methods and supporting references is provided in Table 3.

**TABLE 3:** Summary of experimental evaluation methods used in smart hydrogel‐based MI studies.

Evaluation category	Common methods reported	References
Physicochemical characterization	SEM, TEM, AFM, FTIR, ^1H NMR, UV–vis, Raman spectroscopy, DLS, rheology, swelling analysis, degradation studies, mechanical testing (tensile/compression), zeta potential, XPS, EDS	[5, 6, 31, 46–72, 77–85]
Cell viability and biocompatibility evaluation	CCK‐8, MTT, live/dead staining, cytocompatibility assays, LDH assay, flow cytometry	[5, 6, 31, 46, 48–72, 77–85]
Oxidative stress and antioxidant assessment	DCFH‐DA, DHE staining, DPPH assay, ROS scavenging assays, H_2_O_2_ stimulation models, mitochondrial function assays	[5, 31, 48, 49, 52, 54, 58, 59, 61–64, 67–70, 72, 78, 80, 81, 83–85]
Angiogenesis and regenerative evaluation	Tube formation assays, scratch/migration assays, CD31/VEGF analysis, qRT‐PCR, Western blot, ELISA, immunofluorescence	[5, 6, 31, 46–49, 52–55, 58–72, 77–85]
Molecular and cellular mechanism analysis	qRT‐PCR, Western blot, ELISA, RNA‐seq, immunofluorescence, immunohistochemistry	[5, 6, 31, 46–49, 52–55, 58–72, 77–85]
In vivo cardiac repair evaluation	Echocardiography, H&E staining, Masson’s trichrome staining, Sirius Red staining, TTC staining, TUNEL assay, IHC/IF, infarct analysis	[5, 6, 31, 46–49, 52–55, 57–72, 77–85]
Electrophysiological and functional integration assessment	ECG, electrical conductivity measurements, electrophysiological mapping, arrhythmia testing	[5, 48, 58, 64, 65, 69, 71, 84]
Biodistribution and safety assessment	Cy5 tracking, HPLC analysis, liver/kidney function tests, sterility testing	[5, 6, 46, 64, 67, 71, 77, 85]

*Note:* DCFH‐DA, 2′,7′‐dichlorofluorescin diacetate; DHE, dihydroethidium; DPPH, 2,2‐diphenyl‐1‐picrylhydrazyl; ECG, electrocardiogram; EDS, energy‐dispersive X‐ray spectroscopy; ELISA, enzyme‐linked immunosorbent assay; H&E, hematoxylin and eosin staining; H_2_O_2_, hydrogen peroxide; IF, immunofluorescence; IHC, immunohistochemistry; MTT, 3‐(4,5‐dimethylthiazol‐2‐yl)‐2,5‐diphenyltetrazolium bromide assay; TUNEL, terminal deoxynucleotidyl transferase dUTP nick end labeling; UV–vis, ultraviolet–visible spectroscopy.

Abbreviations: AFM, atomic force microscopy; CCK‐8, Cell Counting Kit‐8; CD31, cluster of differentiation 31; DLS, dynamic light scattering; FTIR, Fourier transform infrared spectroscopy; HPLC, high‐performance liquid chromatography; LDH, lactate dehydrogenase assay; NMR, nuclear magnetic resonance; qRT‐PCR, quantitative reverse transcription polymerase chain reaction; RNA‐seq, RNA sequencing; ROS, reactive oxygen species; SEM, scanning electron microscopy; TEM, transmission electron microscopy; TTC, triphenyltetrazolium chloride; VEGF, vascular endothelial growth factor; WB, Western blot; XPS, X‐ray photoelectron spectroscopy.

#### 3.3.4. Assessment Time Point

Follow‐up durations varied across the included studies depending on the type of evaluation performed. In vitro assessments were generally conducted at 1, 3, and 7 days, with the longest follow‐up reported at 8 weeks (56 days) by Li et al. [57], which included in vitro assessments at 1, 4, 7, and 8 weeks. For in vivo evaluations, follow‐up periods were typically short‐term, most frequently at 7, 14, or 28 days post‐treatment, with the longest observed at 8 weeks in Peng et al. [55].

### 3.4. Applications

As all included studies were conducted in preclinical models, the reported findings reflect the therapeutic potential of smart hydrogels in MI repair rather than clinical efficacy. The therapeutic effects identified across the included studies are summarized in Figure 4. Across in vivo studies, smart hydrogel interventions improved cardiac outcomes through five major functional roles: angiogenesis and vascularization, antifibrotic and antiremodeling effects, immunomodulation, cytoprotection and antiapoptotic activity, and electrophysiological integration (Table 4).

**FIGURE 4:**
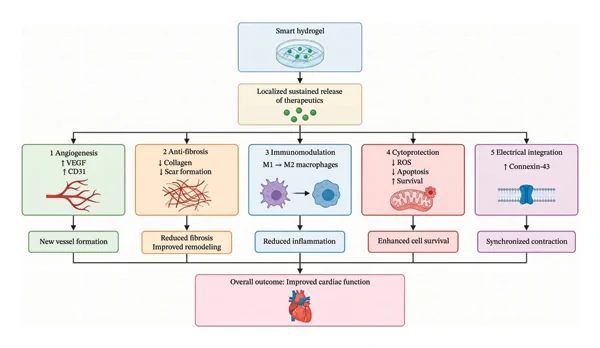
Therapeutic effects of smart hydrogels in myocardial infarction therapy. Initial schematic generated using ChatGPT (OpenAI; accessed 2026), followed by AI‐assisted regeneration and refinement using BioRender. The final figure reviewed and refined by the authors for scientific accuracy. Created in BioRender. Ahadzi, E. (2026) https://BioRender.com/8p1iugc.

**TABLE 4:** Applications of smart hydrogels in MI.

Potential applications	Key functional outcomes	References
Angiogenesis and vascularization	↑ Angiogenesis; ↑ vascularization; ↑ vessel density; ↑ blood flow and tissue perfusion; ↑ vessel network formation; ↑ angiogenic marker expression; neovascularization	[1, 3, 5, 23, 25, 31, 32, 41, 47, 48, 51–55, 58, 60, 62–66, 68–72, 78–85]
Antifibrotic and antiremodeling activity	↓ Collagen deposition; ↓ cardiac remodeling; ↓ myocardial fibrosis; ↑ ventricular wall thickness; restored cardiac structure	[1, 3, 5, 6, 31, 46, 48, 52, 53, 55, 58–61, 63–72, 77–79, 81, 84, 85]
Immunomodulation and anti‐inflammation	M2 macrophage polarization; ↓ inflammation; ↓ early and chronic immune responses; ↓ MMP‐2/9 activity	[5, 6, 31, 32, 54, 59, 61–69, 71, 78–80, 83–85]
Cytoprotection and antiapoptosis	ROS scavenging; ↓ apoptosis; mitochondrial protection; ↑ cell retention and survival; ↓ infarct size; ↑ ventricular function	[3, 5, 6, 31, 32, 41, 46, 48, 50, 54–56, 58–70, 78–80, 82, 83, 85]
Electrophysiological integration	↑ Electrical conduction; ↑ signal velocity; gap junction formation; ↑ connexin‐43 expression; ↓ arrhythmias; ↑ synchronous contraction	[3, 5, 31, 49, 50, 58, 64, 65, 69–72]

Angiogenesis and vascularization (35 studies) and cytoprotection/antiapoptosis (31 studies) were the most frequently reported effects, with enhanced vascular formation associated with CD31/VEGF upregulation and improved cardiomyocyte survival linked to ROS regulation and mitochondrial protection. Antifibrotic effects were reported in 30 studies, with reduced collagen deposition and myofibroblast activation contributing to preservation of ventricular structure. Immunomodulatory effects were observed in 22 studies, primarily through promotion of reparative M2 macrophage polarization, whereas electrophysiological integration, reported in 12 studies, supported improved electrical conduction, gap junction formation, and connexin‐43 (Cx43) expression.

Beyond acute repair, these responsive hydrogels distinguished themselves from passive scaffolds by creating active biofunctional niches that enhance cardiomyocyte alignment [57] and stem cell retention [56]. These multifunctional properties support broader regenerative processes by integrating structural, biochemical, and electrical cues within the injured myocardium [1, 3, 41].

### 3.5. Challenges of Smart Hydrogels in MI

Few studies explicitly reported their study limitations [46, 47, 52, 54, 57, 64, 71, 72, 84]. Reported limitations were categorized into four major domains: biomolecular targeting, material design, methodological limitations, and translational barriers.

The most prominent limitations involved difficulties in achieving precise biomolecular targeting. One study reported limited substrate specificity in MMP‐responsive systems, particularly for MMP‐2/9‐targeted hydrogels, which exhibited suboptimal discrimination between pathological and homeostatic proteolytic activity [52]. Additionally, the temporal mismatch between hydrogel activation and disease progression represented a critical barrier, as MMP‐2/9 activity peaks only briefly in the early post‐MI period [71]. Another study also highlighted an incomplete understanding of the molecular signaling pathways governing post‐MI remodeling, which limited the rational design of more selective biomolecular triggers [72].

Material‐related constraints were also frequently noted. The use of single‐material hydrogels, such as collagen‐only formulations, limited mechanical tunability and biofunctionality in certain systems [52]. Additional issues included poor elasticity and rapid degradation, which compromised structural stability and therapeutic persistence in vivo [57]. Wang et al. further reported that large injection volumes produced unintended mechanical stretching of myocardial tissue, contributing to an undesirable increase in ventricular size [46]. In parallel, Garbern et al. demonstrated that uncontrolled extracellular microenvironments within certain hydrogel systems disrupted pro‐angiogenic signaling and hindered neovascularization [47].

Methodological constraints further impacted the interpretability of findings [72]. The use of immortalized cell lines and immediate post‐MI hydrogel injection has been noted to reduce the clinical relevance of experimental models [54]. In addition, the duration of postinjection inflammatory responses was reported to be uncertain [47]. Zheng et al. also noted the lack of suitable intraoperative imaging systems for hydrogel delivery during open‐heart procedures [64].

Translational limitations were also identified across several studies. Some hydrogels were reported as unsuitable for patients with comorbid conditions such as hormonal dysregulation, peripheral arterial disease, or cancer [52]. Yang et al. emphasized the lack of large‐animal model validation and the reliance on open‐chest surgical delivery methods [84]. In addition, Zheng et al. reported that the absence of minimally invasive catheter‐based injection technologies remains a major obstacle to translating hydrogel therapies into routine clinical practice [64]. A consolidated summary of these limitations is presented in Table 5.

**TABLE 5:** Thematic categorization of challenges.

Challenge	Key challenges reported	References
Biomolecular targeting	Limited specificity to MMP‐2/9; MMP2/9 activity peaks shortly after MI; unclear signaling pathways; limited stimuli response	[32, 52, 71, 72]
Material design	Use of collagen as a sole biomaterial; fast degradation; needs improved elasticity; retention of large hydrogel plugs leading to distension and unstable microenvironment; immunogenic risks; poor cell infiltration complex manufacturing	[3, 32, 46, 47, 52, 57]
Methodological limitations	Inadequate evaluation methods and timing; short‐term functional assessment; immediate injection after MI; use of immortalized cells; lack of imaging devices	[23, 41, 47, 52, 54, 64, 72]
Translational barriers	Unsuitability for MI patients with certain conditions; unclear chronic inflammatory effects; lack of minimally invasive catheter technology; lack of large‐animal studies; incompatible delivery method	[1, 23, 41, 47, 52, 64, 84]

### 3.6. Future Perspectives

Several thematic future directions emerged across the included studies and have been grouped into subthemes (Table 6).

**TABLE 6:** Thematic categorization of future perspectives.

Sub‐theme	Focus area	References
Translational potential	Validation in large‐animal models; scaling up production; minimally invasive delivery; testing in patients with comorbidities; long‐term inflammation evaluation; use of catheter technology; potential clinical application for MI therapy	[1, 3, 5, 23, 31, 41, 46–49, 51, 52, 54, 55, 57–60, 62, 63, 65–72, 77, 82–85]
Mechanistic understanding and biological insight	Immune and microenvironment modulation; multimodal hydrogel repair; Janus hydrogel adhesion‐adaptive mechanisms; hydrogel–cell interactions	[3, 5, 32, 41, 48, 58, 72]
Design optimization	Tuning responsiveness to MI stimuli; structural refinement; exploring alternative biomaterials; optimizing peptide delivery; optimization of injection volume and site	[1, 3, 23, 31, 32, 46, 52, 64, 67]
Expanded applications and combination therapies	Applications to other cardiovascular or electrically responsive tissues; co‐delivery of therapeutics, including small interfering RNA (siRNA), microRNA (miRNA), exosomes, and metformin	[1, 3, 23, 31, 32, 46, 49, 50, 60, 69, 70, 80]

A consistent recommendation across primary studies was the need for extended follow‐up periods to better evaluate long‐term functional recovery, with Garbern et al. specifically advocating for assessments beyond 28 days [47]. A major translational priority identified was the shift from invasive open‐chest delivery toward minimally invasive administration, with emerging research highlighting endoscopic approaches [5] and catheter‐based systems as the most feasible routes for clinical implementation [64, 72, 84]. Additionally, forward‐looking strategies included the proposal by Hu et al. to scale up hydrogel production to meet eventual clinical manufacturing requirements [79]. Fan et al. further emphasized the importance of evaluating therapeutic efficacy in patients with comorbidities, such as cancer, to ensure clinical applicability across diverse patient populations [52].

Further mechanistic exploration was also recommended across several studies. Qiu et al. emphasized the importance of advancing understanding of immune and microenvironmental modulation in hydrogel‐mediated cardiac repair [5], while Hassan et al. called for deeper investigation into multimodal hydrogels that integrate multiple therapeutic functions [48]. He et al. proposed examining molecularly encoded adhesion‐adaptive mechanisms in Janus hydrogels to improve dynamic tissue interfacing [58]. Additional studies highlighted the need to clarify biomaterial interactions with primary cardiomyocytes and fibroblasts [72].

Design‐related future directions focused on structural refinement of hydrogel architectures [31] and exploration of alternative biomaterials to overcome limitations associated with collagen‐based systems [52]. Wang et al. also recommended optimizing peptide sequences to enhance specificity and responsiveness to MMP activity, particularly for MMP‐targeted delivery systems [46].

Expanding therapeutic indications beyond MI was proposed in five studies, suggesting applicability to other cardiovascular tissues and electrically responsive organs [49, 50, 60, 70, 80]. Several studies additionally supported the development of combination therapies, including co‐delivery of pharmacological agents, siRNA/miRNA, exosomes, metformin, and other synergistic components to amplify regenerative efficacy [31, 46, 70, 79].

## 4. Discussion

### 4.1. Applications of Smart Hydrogels in MI Therapy

#### 4.1.1. Hydrogel Material Platforms

Smart hydrogels have emerged as versatile platforms for MI repair, offering diverse mechanistically distinct strategies to address the structural deterioration and cellular dysfunction that characterize postinfarct remodeling. Functionally, these hydrogels provide temporary mechanical reinforcement, reducing ventricular wall stress and limiting pathological dilation, thereby supporting more favorable remodeling trajectories.

Natural polymer‐based hydrogels (e.g., collagen, gelatin) inherently promote cell adhesion, proliferation, and biointegration, owing to their biochemical similarity to native ECM components. However, their clinical utility can be constrained by limited mechanical robustness, rapid degradation, and variable batch‐to‐batch consistency. In contrast, synthetic hydrogels offer precise tunability in stiffness, degradation kinetics, and crosslinking density, enabling more controlled and sustained mechanical support. These systems often require functionalization with peptides, growth factors, or conductive additives to improve cellular interactions and electrophysiological integration. Hybrid hydrogels strategically combine natural and synthetic components to integrate biological activity with improved structural durability, addressing limitations of either material class alone [87, 88]. However, their increased compositional complexity may introduce challenges related to reproducibility, large‐scale manufacturing, and regulatory translation. Figure 5 summarizes smart hydrogel platforms and design approaches for MI therapy.

**FIGURE 5:**
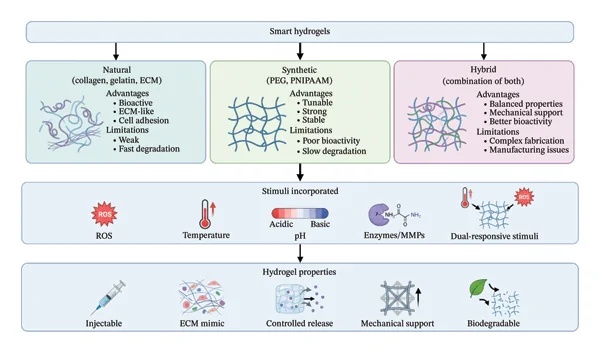
Design strategies for smart hydrogels in myocardial infarction therapy. Initial schematic generated using ChatGPT (OpenAI; accessed 2026), followed by AI‐assisted regeneration and refinement using BioRender. The final figure reviewed and refined by the authors for scientific accuracy. Created in BioRender. Ahadzi, E. (2026) https://BioRender.com/71xeuux.

#### 4.1.2. Stimulus‐Responsive Hydrogel Systems

Beyond providing mechanical support, smart hydrogels exploit microenvironmental cues to achieve more targeted therapeutic effects. ROS‐responsive systems can reduce oxidative stress and inflammation, thereby promoting angiogenesis and preserving myocardial tissue [90]. However, their performance depends on the dynamic ROS microenvironment within injured tissue, where excessive ROS may sustain inflammatory signaling. While these systems offer high biological specificity, achieving reliable ROS‐responsive behavior remains challenging due to the complexity of designing stable and responsive chemistries [91–93].

Temperature‐sensitive hydrogels enable minimally invasive delivery through rapid sol–gel transition at physiological temperature, providing structural support to the injured myocardium. Their simple fabrication and injectability enhance their translational potential; however, their limited adaptability to the dynamic conditions of injured tissues may restrict their ability to provide disease‐specific regulation [94, 95].

pH‐responsive hydrogels preferentially release therapeutic agents within the acidic microenvironment of ischemic myocardium, improving cell survival and vascularization. Nevertheless, because the pH difference between healthy and infarcted myocardium is relatively modest and evolves over time, pH alone may provide limited release selectivity. Optimizing crosslink chemistry, network structure, and degradation kinetics is therefore essential to achieve sustained, stage‐appropriate delivery while minimizing premature release [96, 97].

Enzyme‐responsive hydrogels, particularly those targeting MMP activity after MI, align therapeutic release with ECM remodeling, thereby reducing scar formation and supporting antifibrotic repair. However, variations in enzyme activity across different patients and disease stages may influence hydrogel degradation and drug‐release behavior. Furthermore, incorporating enzyme‐responsive components increases design complexity and presents challenges in maintaining consistent fabrication, scalability, and material performance [98–100].

More recently, dual‐responsive hydrogels, previously validated in other biomedical applications, have demonstrated the capacity to integrate multiple stimuli, enabling multistage, on‐demand therapeutic release and improved regulation of inflammation, fibrosis, and tissue remodeling [101–103]. However, the combination of multiple responsive mechanisms increases design complexity and requires careful optimization of degradation behavior to maintain long‐term stability and therapeutic effectiveness [104, 105].

Overall, these stimulus‐responsive systems demonstrate a trade‐off between biological specificity and engineering feasibility, where greater responsiveness often requires more sophisticated material design and characterization strategies [106, 107]. Temperature‐responsive systems provide advantages in fabrication simplicity and practicality, whereas ROS‐, pH‐, enzyme‐, and dual‐responsive platforms offer enhanced disease‐specific control but require further optimization of responsiveness, degradation behavior, and manufacturing consistency.

#### 4.1.3. Therapeutic Cargo Delivery Strategies

The therapeutic potential of these hydrogels also depends on the incorporated bioactive agents, each of which acts through distinct mechanisms.

Cell‐based therapies can promote myocardial repair through cardiomyocyte replacement and paracrine signaling that stimulates angiogenesis, reduces inflammation, and supports tissue remodeling. However, their clinical potential remains limited by poor survival and engraftment within the infarcted myocardium due to hypoxia, oxidative stress, and inflammatory conditions [108]. EVs provide proangiogenic and anti‐inflammatory benefits with lower immunogenicity, although variability in EV cargo composition presents reproducibility challenges [109, 110]. Small molecules and drugs modulate signaling pathways and inflammatory responses but often require controlled delivery strategies due to rapid clearance [111]. Protein‐based therapeutics enhance angiogenesis and cell survival but are limited by structural instability and degradation [112]. Genetic materials (e.g., siRNA, miRNA, plasmids) enable long‐term regulation of fibrosis and ECM remodeling, while nanoparticles enhance delivery and provide additional functions, although safety and biocompatibility require further evaluation [113, 114].

Each cargo type presents distinct advantages and limitations; therefore, integrating multiple agents within smart hydrogel systems may leverage their complementary strengths to achieve enhanced therapeutic effects for myocardial repair.

Taken together, these systems provide multifunctional platforms that integrate responsive behavior with targeted cargo delivery. Hybrid and dual‐responsive designs offer promising strategies for balancing structural stability and controlled therapeutic regulation, although further optimization might be needed to improve translational potential.

### 4.2. Challenges in Translating Smart Hydrogel‐Based MI Therapy

#### 4.2.1. Preclinical Model Limitations

Despite promising preclinical results, the translation of smart hydrogel therapies for MI remains challenged by several scientific, methodological, and practical limitations. A major challenge is the heavy reliance on small‐animal models, particularly rodents. SD rats are commonly used due to their larger heart size [115], whereas Fischer rats are chosen for their more stable immune and stress responses [116]. C57BL/6J mice are often selected for their genetic traceability and the availability of transgenic lines [117]. However, these models do not fully replicate human cardiac physiology, infarct complexity, or clinically relevant comorbidities such as diabetes and hypertension [118, 119]. Furthermore, the frequent use of male‐only cohorts may introduce potential sex‐related bias, as differences in cardiac remodeling, immune activation, and biomaterial responses may influence therapeutic outcomes.

Compounding these limitations, small‐animal studies also commonly involve short follow‐up periods, often spanning only a few days to several weeks after infarction. These limited observation windows restrict assessment of long‐term outcomes, including ventricular remodeling, scar maturation, sustained functional recovery, chronic immune responses, arrhythmogenic risk, and hydrogel degradation behavior [120]. Such limitations may overlook delayed immune responses, degradation‐related changes, and long‐term tissue interactions.

#### 4.2.2. Delivery Challenges

Hydrogel delivery also poses substantial translational challenges. Most preclinical works rely on open‐chest surgical administration, which is not feasible for routine clinical application. Intramyocardial injection remains the dominant approach [121]; however, limited hydrogel distribution within myocardial tissue may affect electrical integration and increase arrhythmogenic risk [122].

Alternative delivery routes, including subcutaneous and intramuscular administration, have been explored to improve distribution. Subcutaneous delivery allows repeated administration but is limited by slower absorption [123], whereas intramuscular delivery may provide improved vascular access and uptake [124]. However, these approaches provide less precise targeting than intramyocardial delivery. The absence of minimally invasive delivery systems, such as catheter‐based approaches, remains a translational barrier due to challenges in achieving precise hydrogel localization while preserving therapeutic function.

#### 4.2.3. Material Safety, Biocompatibility, and Sterilization Constraints

Material‐related safety concerns also limit translational potential. Hydrogels must retain structural integrity and bioactivity within the dynamic mechanical environment of the myocardium; however, excessive injection volumes may mechanically strain the ventricular wall and adversely affect remodeling outcomes [6]. In addition, long‐term biocompatibility and immune responses remain insufficiently characterized, as chronic inflammation may disrupt ECM organization and angiogenesis [47].

Furthermore, sterilization requires careful optimization to eliminate microbial contamination without compromising hydrogel structure, mechanical properties, degradation behavior, or bioactivity. Conventional approaches, including autoclaving, ethylene oxide, and gamma irradiation, may alter polymer networks or damage encapsulated therapeutic cargos [125, 126], potentially affecting hydrogel performance and clinical translation.

#### 4.2.4. Manufacturing Scalability and Regulatory Translation

Scalability is also a major obstacle to clinical translation. Hydrogels optimized under controlled laboratory conditions may behave differently during large‐scale production, where batch‐to‐batch variability can affect consistency, reproducibility, and therapeutic performance [127–129]. Furthermore, the lack of standardized manufacturing processes, robust quality control (QC), and good manufacturing practice (GMP)‐compliant production further complicates large‐scale manufacturing and regulatory approval.

Regulatory evaluation is further complicated by the multifunctional nature of smart hydrogels, which often integrate responsive networks with therapeutic components [130]. The interactions between these components can affect cargo stability, release behavior, and overall therapeutic performance, making it more complex to assess factors such as biomaterial degradation, long‐term safety, product quality, and manufacturing consistency [131].

### 4.3. Future Perspectives on Smart Hydrogel Design and Clinical Translation

#### 4.3.1. Improving Preclinical Evaluation

Addressing the current limitations of smart hydrogel therapies for MI will require advances in preclinical models, material design, and delivery strategies. Future research could benefit from large‐animal models, such as pigs, which more closely mimic human myocardial size, anatomy, and infarct heterogeneity [132]. These models could enable more comprehensive evaluation of therapeutic durability, including chronic inflammatory responses, vascular remodeling, and electrical integration over extended periods. Including both male and female subjects is important, as it improves translational relevance and helps account for sex‐specific differences in cardiac remodeling and biomaterial responses.

#### 4.3.2. Expanding Biological Responsiveness of Smart Hydrogels

Beyond improving preclinical evaluation, future studies should also expand the range of biological stimuli targeted by smart hydrogels. MMP‐sensitive hydrogels, particularly targeting MMP‐2 and MMP‐9, are widely explored due to their rapid upregulation after MI and their role in ECM remodeling [103]. Other isoforms, including MMP‐1, ‐3, ‐7, and ‐14 [52, 74, 133], may provide additional opportunities for developing more adaptive responsive hydrogels, as these enzymes are involved in broader inflammatory and remodeling processes [134]. Enzymes involved in postinfarction remodeling, such as myeloperoxidase (MPO) [135], may also serve as potential responsive targets. Additionally, incorporating additional physiological signals, such as glucose fluctuations, lactate changes, mechanical stress, and ionic variations, may better replicate the dynamic cardiac microenvironment. Moreover, immune and microenvironmental modulation represents an important direction, enabling hydrogels to modulate interactions among immune cells, cardiomyocytes, and fibroblasts to support tissue repair and remodeling.

#### 4.3.3. Advanced Delivery Strategies

Advancements in delivery strategies will also be important for improving clinical feasibility. Intramyocardial delivery may be adapted through minimally invasive approaches, including catheter‐based and endoscopic systems. Catheter‐based transendocardial intramyocardial injection enables localized hydrogel delivery under real‐time imaging guidance [136]. This approach reduces the need for open‐heart surgery while allowing targeted delivery to the infarcted region. Further refinement of these platforms may improve hydrogel placement, reduce procedural invasiveness, and preserve therapeutic function during administration. Similarly, endoscopic approaches provide direct visualization of the heart surface [137], enabling minimally invasive hydrogel placement with improved accuracy and reduced surgical exposure. Achieving an optimal balance between hydrogel retention and controlled release will be key to maximizing clinical effectiveness. Key considerations for clinical translation are shown in Figure 6.

**FIGURE 6:**
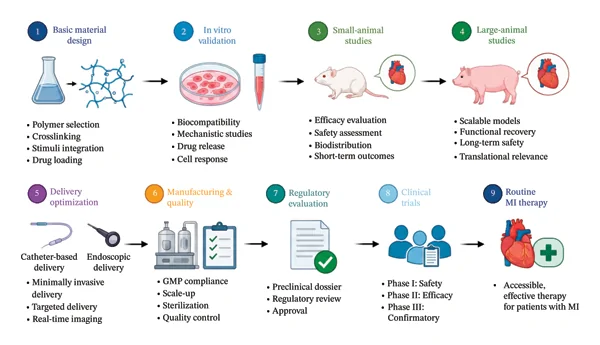
Translational roadmap for smart hydrogel‐based myocardial infarction therapy. Initial schematic generated using ChatGPT (OpenAI; accessed 2026), followed by AI‐assisted regeneration and refinement using BioRender. The final figure reviewed and refined by the authors for scientific accuracy. Created in BioRender. Ahadzi, E. (2026) https://BioRender.com/nhfrhr0.

#### 4.3.4. Manufacturing and Regulatory Readiness

Addressing scalability and regulatory challenges will also be critical for advancing smart hydrogels toward clinical use. Moving from laboratory‐scale production to clinical use will require manufacturing approaches that ensure consistent hydrogel properties and therapeutic performance. Future efforts should focus on robust quality control strategies, GMP‐compliant production, and sterilization methods that preserve hydrogel integrity, mechanical properties, and bioactive cargo stability. In addition, standardized manufacturing processes and careful evaluation of safety, efficacy, and product consistency will be important for meeting regulatory requirements and supporting clinical adoption.

#### 4.3.5. AI‐Assisted Hydrogel Design and Personalized Therapies

Advances in computational approaches are creating new opportunities for smart hydrogel development. AI and machine‐learning tools could help guide hydrogel design by predicting how properties such as mechanical strength, degradation, stimulus responsiveness, and bioactivity affect therapeutic outcomes. This may reduce reliance on trial‐and‐error approaches and accelerate the optimization of hydrogel systems.

Computational approaches may also support more personalized regenerative therapies by enabling hydrogel designs tailored to individual patient characteristics, including infarct features, cardiac function, and biological responses. Combining computational predictions with experimental validation and advanced fabrication strategies could improve the precision and effectiveness of smart hydrogel‐based therapies.

#### 4.3.6. Expanding Therapeutic Applications

Beyond MI, these platforms hold potential for broader cardiovascular applications and other electrically active tissues. They could be engineered to support conduction tissue regeneration, vascular repair, or arrhythmia management, leveraging their responsiveness to mechanical, electrical, and biochemical cues. By extending hydrogel therapies to these contexts, they may evolve beyond MI‐focused interventions into broader regenerative platforms capable of repairing a wide range of cardiovascular and electromechanically active tissues, thereby significantly broadening their clinical impact.

### 4.4. Study Limitations

While these findings offer valuable insights, this scoping review has some limitations. Only a few studies explicitly discussed their limitations, which may lead to an underrepresentation of the challenges faced. Second, all included studies were conducted at the preclinical level, with no clinical trials reported. This limits the direct applicability of the findings to real‐world patient care and clinical practice. Third, most studies were geographically concentrated in China, which may limit the generalizability of results to other regions. Finally, book chapters and grey literature were excluded, which could have led to the omission of emerging insights or unpublished innovations not yet represented in peer‐reviewed journals.

## 5. Conclusion

Smart hydrogels represent a rapidly evolving platform in MI therapy, offering integrated mechanical and biochemical support to restore cardiac structure and function. Advances in dual‐responsive systems, hybrid polymer networks, and combinatorial delivery strategies have significantly enhanced their capacity to promote angiogenesis, reduce fibrosis, and modulate postinfarction inflammation. Despite these promising outcomes, several challenges still limit clinical translation. These include dependence on small‐animal models, short follow‐up periods, limited minimally invasive delivery options, long‐term biosafety concerns, sterilization challenges, and difficulties with scalable manufacturing and regulatory approval. Future research should focus on long‐term studies using clinically relevant models, improved catheter‐based delivery approaches, better understanding of hydrogel–tissue interactions, and standardized manufacturing processes. Collectively, addressing these challenges will help move smart hydrogels from experimental platforms toward next‐generation therapies for myocardial repair and cardiac regenerative medicine.

## Author Contributions

Conceptualization: Eyram Ahadzi, Otema Darfour Manu, and Prince Ebenezer Adjei; methodology, investigation, and data curation: Eyram Ahadzi and Otema Darfour Manu; visualization: Eyram Ahadzi; writing–original draft preparation: Eyram Ahadzi; supervision: Prince Ebenezer Adjei; writing–review and editing: Ruth Antwi‐Baah, Henry Agbe, and Prince Ebenezer Adjei.

## Funding

No funding was received for this manuscript.

## Conflicts of Interest

The authors declare no conflicts of interest.

## Supporting Information

Additional supporting information can be found online in the Supporting Information section.

## Supporting information


**Supporting Information** The supporting information included with this manuscript is as follows: Supporting 1: The Preferred Reporting Items for Systematic Reviews and Meta‐Analyses extension for Scoping Review (PRISMA‐ScR) checklist was used for this scoping review. It ensured adherence to PRISMA‐ScR guidelines and provided a clear record of all methodological steps, including screening, eligibility assessment, and data extraction. Supporting 2: Detailed search strategies and Boolean queries used to retrieve relevant literature from PubMed, Scopus, and ScienceDirect. This includes the exact syntax, keywords, and search combinations applied in each database to ensure a comprehensive, transparent, and reproducible search process.

## Data Availability

Data sharing is not applicable to this article, as no original datasets were generated.
