# Supplementary material for: Advances in Smart Hydrogels for Myocardial Infarction Therapy: A Scoping Review of Applications, Challenges, and Future Perspectives
Source: Int J Biomater. 2026 Oct 18;2026:7925127. doi: 10.1155/ijbm/7925127 (PMC13678413; doi:10.1155/ijbm/7925127)
Supplement: Supplementary file 1 [file IJBM-2026-7925127-s001.zip › Supplementary 2.docx]

**Supplementary 2**

**SEARCH STRATEGY**

**DATABASES SEARCHED:** *PUBMED, Scopus, Science Direct*

**PUBMED**

**#1 Myocardial Infarction Therapy OR. Myocardial* OR Cardiac Regeneration OR Heart Tissue Regeneration OR Myocardial Tissue Repair**

**#2 Myocardial Infarction OR Heart Attack OR Acute Myocardial Infarction OR Cardiac Injury**

**#3 Smart Hydrogels OR Stimuli-Responsive Hydrogels OR Intelligent Hydrogels**

**#4 Application* OR Current Applications OR Clinical Applications OR Therapeutic Applications**

**#5 Challenges OR Barriers OR Limitations OR Issues OR Problems**

**#6 Future Perspectives OR Emerging Trends OR Innovations OR Advancements**

**#7 (#1 OR #2)**

**#8 (#4 OR #5 OR #6)**

**#9 (#3 AND #7 AND #8)**

**SCOPUS**

("Myocardial Infarction Therapy" OR Myocardial* OR "Cardiac Regeneration" OR "Heart Tissue Regeneration" OR "Myocardial Tissue Repair" OR "Myocardial Infarction" OR "Heart Attack" OR "Acute Myocardial Infarction" OR "Cardiac Injury")

AND

("Smart Hydrogel*" OR "Stimuli-Responsive Hydrogel*" OR "Intelligent Hydrogel*")

AND

(Application* OR "Clinical Application*" OR "Therapeutic Application*" OR Challenge* OR Barrier* OR Limitation* OR Issue* OR Problem* OR "Future Perspective*" OR "Emerging Trend*" OR Innovation* OR Advancement*)

**SCIENCE DIRECT**

("Myocardial Infarction" OR "Heart Attack" OR "Acute Myocardial Infarction") AND ("Smart Hydrogels" OR "Stimuli-Responsive Hydrogels" OR “Intelligent Hydrogels”) AND ("Applications" OR "Challenges" OR "Innovations")
